# Effect of histamine‐receptor antagonism on the circulating inflammatory cell and cytokine response to exercise: A pilot study

**DOI:** 10.14814/phy2.15936

**Published:** 2024-02-02

**Authors:** Matthew R. Ely, Joshua E. Mangum, Karen Wiedenfeld Needham, Christopher T. Minson, John R. Halliwill

**Affiliations:** ^1^ Department of Human Physiology University of Oregon Eugene Oregon USA

**Keywords:** histamine, antihistamine, exercise, inflammation, leukocytosis

## Abstract

The purpose of this study was to gain insight into histamine's role in the exercise inflammatory response and recovery from exercise. To explore this, young healthy participants (*n* = 12) performed 300 eccentric leg extensions under control (Placebo) versus histamine H_1_ and H_2_ receptor antagonism (Blockade) in a randomized cross‐over study. Circulating leukocytes and cytokines were measured for 72 h after exercise. Circulating leukocytes were elevated at 6 and 12 h after exercise (*p* < 0.05) with the peak response being a 44.1 ± 11.7% increase with Blockade versus 13.7 ± 6.6% with Placebo (both *p* < 0.05 vs. baseline, but also *p* < 0.05 between Blockade and Placebo). Of the cytokines that were measured, only MCP‐1 was elevated following exercise. The response at 6 h post‐exercise was a 104.0 ± 72.5% increase with Blockade versus 93.1 ± 41.9% with Placebo (both *p* < 0.05 vs. baseline, *p* = 0.82 between Blockade and Placebo). The main findings of the present investigation were that taking combined histamine H_1_ and H_2_ receptor antagonists augmented the magnitude but not the duration of the increase of circulating immune cells following exercise. This suggests histamine is not only exerting a local influence within the skeletal muscle but that it may influence the systemic inflammatory patterns.

## 1 INTRODUCTION

Histamine concentrations increase within contracting skeletal muscle by mast cell degranulation and through de novo production by the enzyme histidine decarboxylase (Romero, McCord, et al., 2016b). Work from our lab has shown a 3‐fold increase in histamine concentration during 60 min of moderate intensity exercise (Romero, McCord, et al., 2016b). Although the physiological significance of the exercise induced elevation in histamine is unknown, our lab has demonstrated that systemically blocking histamine H_1_ and H_2_ receptors blunts sustained post‐exercise vasodilation and reduces skeletal muscle blood flow in the initial hours following exercise (McCord et al., 2006; McCord & Halliwill, 2006). Additionally, recent work from our laboratory has shown that blocking histamine receptors during aerobic exercise alters the expression of ~800 protein coding‐genes associated with the exercise responsome (Ely, 2020; Romero et al., 2018; Romero, Hocker, et al., 2016a). Among the altered protein coding genes are the genes for interleukin 6 (IL‐6), monocyte chemoattractant protein‐1 (MCP‐1), interleukin 1 receptor‐like 1 (IL1RL1) and others involved with immune signaling pathways (Romero et al., 2018; Romero, Hocker, et al., 2016a).

Outside of exercise, histamine plays a major role in the generalized inflammatory response. For example, following tissue damage there is an increase in the concentration of histamine in the surrounding area (Majno et al., 1961). This histamine stimulates the production of chemokines, increases capillary permeability via margination of capillary endothelial and pericyte cells, and increases the expression of adhesion molecules on endothelial cells (Beer et al., 1984; Jutel et al., 2001; Packard & Khan, 2003). Together, these actions permit leukocyte infiltration into damaged tissue (chemotaxis) and initiate tissue repair (Beer et al., 1984; Jutel et al., 2002; Packard & Khan, 2003). These lines of research suggest that histamine may be an integral factor linking exercise and immune or inflammatory responses.

Exercise is a physiological stress that causes hormonal and immunological responses similar to physical stressors (e.g., trauma, burn, sepsis) (Pedersen & Hoffman‐Goetz, 2000). Following a single bout of high‐intensity exercise, prolonged endurance exercise, or muscle damaging exercise, there is an increase in the number of circulating white blood cells (leukocytosis) (Campbell & Turner, 2018; Hansen et al., 1991; Natale et al., 2003; Nieman et al., 2005; Peake et al., 2005; Severs et al., 1996; Van Craenenbroeck et al., 2014). This rise in leukocytes appears to peak 3–12 h after exercise cessation and can remain elevated for up to 48 h. The leukocytes are believed to be mobilized out of peripheral tissues including the lymphatic system, bone marrow, and the spleen into the circulation (Severs et al., 1996). In turn, the circulating leukocytes enter the previously active skeletal muscle in the hours to days after exercise cessation (Deyhle et al., 2015; MacIntyre et al., 1996; Peake et al., 2017). Although the inflammatory response may vary based on exercise mode, duration, and intensity, we anticipated that a robust response would occur following exercises that result in muscle ultrastructural damage (Peake et al., 2017; Pyne, 1994). It is possible that exercise induced elevations in skeletal muscle histamine may be a key regulator of the initiation and resolution of an inflammatory response following exercise.

Therefore, the purpose of this study was to gain insight into histamine's role in the exercise inflammatory response and recovery from exercise. To explore the association of histamine and the inflammatory response following exercise, we used two models (1) eccentric muscle contractions and (2) moderate‐intensity aerobic exercise. Specifically, an initial cohort of human subjects performed 300 eccentric leg extensions (Study 1). Subsequently, a second cohort of subjects performed 60 min of cycling exercise at 60% of VO_2peak_ (Study 2). Circulating leukocytes and cytokines were measured periodically for 72 h after eccentric exercise and 48 h after aerobic exercise. Subjects in both cohorts performed exercise in conditions where histamine's effects on H_1_ and H_2_ receptors were antagonized (Blockade) and in a placebo condition (Placebo). It was hypothesized that H_1_ and H_2_ receptor antagonism would alter the inflammatory response to exercise by extending the duration of leukocytosis and reducing the expression of inflammatory cytokines.

## 2 METHODS

These studies were approved by the Institutional Review Board of the University of Oregon. Each volunteer gave written and informed consent prior to participation and the study conformed to the principles of the Declaration of Helsinki.

### 2.1 Subjects

Nineteen healthy volunteers (5 female and 14 male, self‐reported) completed these studies (voluntary response sampling). Subjects were considered sedentary or recreationally active based on exercise habits over the previous 12 months (Kohl et al., 1988) being less than 3 days of exercise/week or less than 90 min/week. Inclusion criteria were age 18–40 and sedentary or recreationally active. Exclusion criteria were medical history of cardiovascular disease, diabetes, autonomic disorders, or asthma, elevated blood pressure, smoking or nicotine use, ongoing medical therapy (other than birth control), pregnant or breastfeeding women, allergies to medications, currently taking NSAIDS or other anti‐inflammatory medications, current OTC/Rx antihistamine use, body mass index >28 kg/m^2^. No subjects were using over the counter or prescription medications at the time of the study, with the exception of oral contraceptives. Female participants were studied during the early follicular phase of their menstrual cycle or during the placebo phase of their oral contraceptive to minimize any potential effects of sex‐specific hormones on muscle damage and immune responses. Demographic and anthropometric measurements (e.g., age, height, weight) were made prior to the initial study day.

### 2.2 Overall experimental design

**FIGURE 1:**
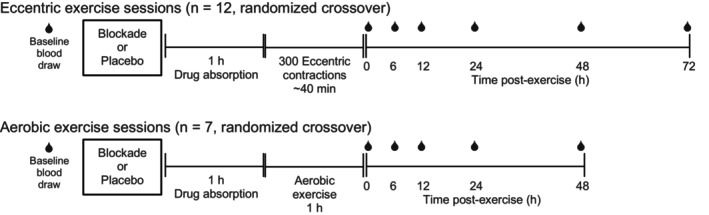
Protocol timelines for the eccentric and aerobic exercise sessions.

This was a randomized placebo‐controlled study with two non‐randomized cohorts of subjects. Subjects in the initial cohort completed two sessions of eccentric exercise (*n* = 12) while subjects in the second cohort completed two sessions of aerobic exercise (*n* = 7). As depicted in Figure 1, within each cohort, subjects were randomized, by a researcher not involved with the data collection, to receive either combined histamine H_1_/H_2_ receptor antagonists (Blockade) or placebo pills (Placebo) in a crossover design. H_1_/H_2_ receptor antagonist or placebo pills were consumed orally with 180 mL of water and subjects were seated for 60 min following consumption, prior to exercise. Venous blood samples were obtained from an antecubital vein prior to drug consumption, immediately following exercise, and 6, 12, 24, 48, and 72 h after eccentric exercise. After aerobic exercise, samples were obtained with the same timing, but only obtained through 48 h, as moderate intensity aerobic exercise was not expected to produce as prolonged a leukocytosis as eccentric exercise (Brenner et al., 1999; Peake et al., 2017). As the length of exercise was not identical in the two protocols, this resulted in the immediate post‐exercise blood sample being obtained approximately 100 min after drug consumption for the eccentric exercise group and 120 min after drug consumption in the aerobic exercise group.

### 2.3 Rigor and reproducibility

A food diary was provided to the subjects prior to the first testing visit. The food diary encompassed the 24‐h prior to the initial exercise as well as the 48–72 h observation period after exercise. Prior to the second testing visit, subjects were provided with their food diary from the first testing visit and asked to mimic food intake (timing, type, and quantity) as close as possible in order to control for potential influences of carbohydrate intake on the inflammatory response or protein intake on skeletal muscle remodeling (Depner et al., 2010). Subjects reported to the laboratory in the morning after an overnight fast and were required to abstain from caffeine, alcohol, and exercise for 24‐h before and for the duration of each observational period. Data collection was briefly disrupted in Study 2 due to a drug recall (described below), and ultimately terminated by pandemic restrictions.

### 2.4 Eccentric exercise

Subjects performed 300 eccentric contractions of the leg extensors with one leg in a protocol modeled after Newham et al., 1983 and MacIntyre et al., 1996 in which 300 eccentric contractions of the quadricep muscles induced a moderate level of muscle damage and caused a local inflammatory response. The angular velocity was set to 60° s^−1^ for set 1 and 110° s^−1^ for set 2 through 10. The resistance force of the dynamometer (Biodex, system 3, Shirley NY) was set at 140% of each subject's maximal isometric force measured at a joint angle of 30° of flexion. Subjects were instructed to resist knee flexion as best as they could. Their leg was then moved passively by the dynamometer back into the extended position. The whole protocol was approximately 40 min in duration. Subjects were block randomized into one of four different testing orders which alternated both the leg to be exercised (left or right) as well as Placebo and Blockade conditions. To avoid possible cross limb (e.g., left to right leg) adaptations that protect muscle from damage/soreness resulting from eccentric contractions, cross‐over trials were separated by at least 30 days. Additionally, measures of isometric strength and blood creatine kinase were obtained pre‐exercise and at 0, 6, 12, 24, 48 and 72 h post‐exercise to confirm muscle damage.

### 2.5 Aerobic exercise

Subjects performed 60‐min of stationary cycling at a power output corresponding to 60% of peak oxygen uptake (VO_2peak_), as determined from a peak oxygen uptake test on a screening visit, as described previously (McCord et al., 2006; McCord & Halliwill, 2006). Exercise at this intensity has been demonstrated to produce a robust elevation in intramuscular histamine and histamine mediated sustained post exercise vasodilatory response (McCord et al., 2006; McCord & Halliwill, 2006; Romero, McCord, et al., 2016b). Heart rate was also measured during aerobic exercise using a three‐lead electrocardiograph (Tango+, SunTech Medical, Raleigh, NC, USA). Placebo and Blockade testing days were separated by at least 30 days. For comparison to earlier studies, a limited set of hemodynamic measurements were made pre‐ and post‐exercise with the subjects in the supine position. Arterial blood pressure was measured in the right arm using an automated sphygmomanometer (Tango+, SunTech Medical, Raleigh, NC, USA). Femoral artery blood flow was measured with a linear‐array ultrasound transducer (9 MHz, Phillips iE33, Andover, MA., USA), using standard methods for quantification (Buck et al., 2014; Romero et al., 2015). Femoral vascular conductance was calculated by dividing femoral blood flow by mean arterial pressures and expressed as mL min^−1^ mmHg^−1^.

### 2.6 Drugs

For both the eccentric exercise and aerobic exercise cohorts, subjects consumed either placebo or combined histamine H_1_/H_2_ receptor antagonists. These consisted of 540 mg of fexofenadine (Kirkland AllerFex, various lots) and 300 mg of ranitidine (Zantac, various lots) for all subjects in the eccentric exercise cohort and five subjects in the aerobic exercise cohort. Due to an FDA recall on ranitidine, 40 mg of famotidine (Pepcid AC, single lot) was substituted for ranitidine in the last two subjects in the aerobic exercise cohort. Oral administration of fexofenadine, a selective H_1_‐receptor antagonist, reaches peak plasma concentrations within 1‐h and has a 12‐h half‐life (Russell et al., 1998). Oral administration of ranitidine, a selective H_2_‐receptor antagonist, reaches peak plasma concentration within 2‐h and has a 3‐h half‐life (Garg et al., 1985). Oral administration of famotidine, a selective H_2_‐receptor antagonist, reaches peak plasma concentration within 2‐h and has a 3‐h half‐life, similar to ranitidine. Others have found that famotidine has a similar impact on post‐exercise vasodilation to what has been found with ranitidine (Van der Stede et al., 2021). This dosage of histamine receptor antagonists results in more than 90% inhibition of histamine H_1_ and H_2_ receptors lasting for 6‐h after administration (Garg et al., 1985). Fexofenadine, ranitidine, and famotidine are not thought to cross the blood–brain barrier or to have sedative effects (Garg et al., 1985; Russell et al., 1998). Importantly, these drugs have no impact on resting blood flow, heart rate, blood pressure, or smooth muscle tone (Ely et al., 2016; McCord et al., 2006; Romero, Hocker, et al., 2016a). The placebos were manufactured by a compounding pharmacy (Creative Compounds, Wilsonville, OR) and contained the inactive ingredients of the fexofenadine and ranitidine tablets. Participants and researchers involved in data collection and analysis were blinded to the administration of placebo and histamine receptor antagonists.

### 2.7 Blood sampling

For each experimental session, a baseline blood draw occurred before consumption of Placebo or Blockade and another blood draw occurred immediately following exercise. Subjects then went about their regular day but returned to the lab at specified times after the completion of exercise to provide additional venous blood samples as noted above. At each time point, a total of 9 mL of venous blood was collected from a superficial vein in the antecubital space into vacutainers (BD Vacutainer, Becton, Dickinson and Company, Franklin Lakes NJ, USA) containing EDTA. A portion of the blood was used for flow cytometry (Gallios, Beckman Coulter Life Sciences, Indianapolis IN, USA) to quantify white blood cell populations (Kaluza, Beckman Coulter Life Sciences, Indianapolis IN, USA) as described below. Additionally, plasma was aliquoted and saved for subsequent analysis of cytokines. In Study 1, plasma was also analyzed for creatine kinase, as a marker of muscle damage, using an activity assay (Sigma‐Aldrich MAK116‐KT, St. Louis MO, USA).

### 2.8 Cell staining protocol for flow cytometry

Cell suspensions were re‐suspended and 1.5 × 10^6^ cells were transferred to individual 2.0 mL microcentrifuge tubes for each staining sample plus the unstained cells. Cells were pelleted at 500G for 5 min. Cells were re‐suspended in 100 μL of staining buffer (PBS + 1% BSA + 10% FBS = Biolegend 420,201 + 50 mL FBS—Gibco 26,140‐129, heat deactivated at 56° for 45 min) plus 10 μL mouse IgG (Jackson Immuno—ChromPure 015‐000‐003). Suspensions were incubated for 30 min at room temperature. Cells were washed with 1 mL of staining buffer, centrifuged and supernatant was discarded. 100 μL of antibody staining cocktail was added to each tube and incubated for 30 min in the dark at room temperature. Then, 1 mL of staining buffer was added to each tube, cells were centrifuged, and supernatant was discarded. Cell pellet was re‐suspended in 500 μL staining buffer and read in a flow cytometer. The following antibodies were used for the leukocyte staining protocol: CD62E (Thermo Fisher Scientific A15436, E‐selectin Monoclonal Antibody [1.2B6], FITC), CD86 (Biolegend 305,406, PE anti‐human CD86), CD309 (Biolegend 359,918, PE/Dazzle™ 594 anti‐human CD309 [VEGFR2]), CD34 (Biolegend 343,520, PerCP anti‐human CD34), CD192 (Biolegend 357,212, PE/Cy7 anti‐human CD192 [CCR2]) CD66B (Biolegend 305,118, APC anti‐human CD66b), CD14 (Biolegend 367,114, Alexa Fluor® 700 anti‐human CD14), CD16 (Biolegend 360,710, APC/Cy7 anti‐human CD16), CD3 (Biolegend 344,824, Pacific Blue™ anti‐human CD3), CD19 (Biolegend 363,036, Pacific Blue™ anti‐human CD19), CD56 (Biolegend 362,520, Pacific Blue™ anti‐human CD56 [NCAM]), CD45 (Thermo Fisher Scientific MHCD4530TR, CD45 Monoclonal Antibody [HI30], Pacific Orange). During cell separation, there were 105,618 ± 7815 singlets counted per time point. Monocyte cells were identified and separated by the CD14, CD66, and CD86 surface markers and sub populations of monocytes were based off CD16 and CD192 surface markers. Monocyte subsets were defined as CD14^++^CD16^−^CCR2^+^ (Mon1, or classical), CD14^++^CD16^+^CCR2^+^ (Mon2, intermediate), and CD14^+^CD16^++^CCR2^−^ (Mon3, non‐classical) neutrophils were identified based off CD66B and CD16 surface markers.

### 2.9 Circulating cytokine analysis

Plasma was also analyzed for inflammatory cytokines via a bead‐based flow cytometry kit (Biolegend 740,809, LEGENDplex Human Inflammation Panel 1, multianalyte flow assay kit). This method of cytokine identification uses beads of varying size coated with antigens specific to cytokines of interest. The panel included markers for IL‐1β, TNF‐α, MCP‐1, IL‐6, IL‐8, IL‐10, and IL‐18. The size and fluorescence of the beads allows for separation and quantification of the cytokine by flow cytometry (Gallios, Beckman Coulter Life Sciences, Indianapolis IN, USA). We were able to run this analysis in all the Study 1 participants and in the first five participants in Study 2 as a batch. The pandemic shut down research before we were able to run a second batch, which would have included plasma samples from the last two participants. If a sample concentration was below the detectable limit, we imputed a value of half the lower limit of detection.

### 2.10 Statistical analysis

Data were checked for normality and statistical inferences were drawn from either paired *t*‐tests (for simple comparisons between Placebo and Blockade conditions) or two‐way repeated‐measures ANOVA with a priori contrasts (for measurements across time and between conditions). Significance was set at *p <* 0.05. All data are presented as means ±95% confidence intervals, except for data characterizing the subjects, which are presented as mean ± SD. (Table 1).

**TABLE 1:** Subject demographic and anthropometric measurements.

	Eccentric exercise	Aerobic exercise
*n*	12 (3 female, 9 male)	7 (2 female, 5 male)
Age (years)	24 ± 3	24 ± 3
Height (m)	1.82 ± 0.12	1.62 ± 0.10
Weight (kg)	78.4 ± 16.4	75.1 ± 9.7
BMI (kg m^−2^)	23.5 ± 3.0	23.2 ± 2.8

*Note*: Values are mean ± SD.

Abbreviations: BMI, body mass index; kg, kilograms; m, meter; n, number.

## 3 RESULTS

### 3.1 Subject characteristics

Subject anthropometric and demographic characteristics are displayed in Table 1. Subject characteristics are similar to those obtained previously in our laboratory in young healthy subjects and are consistent with recreationally active individuals. Blood was not obtained for one subject completing the eccentric exercise at 6 h post‐exercise while in the Placebo and 48 h post‐exercise in the Blockade condition.

### 3.2 Exercise responses

#### 3.2.1 Eccentric

All subjects completed the 300 eccentric contractions. There were no differences in the amount of total work performed between Placebo (64.3 ± 10.7 kNm) and Blockade (62.1 ± 9.4 Nm) over the 10 sets (*p =* 0.32). Prior to the 300 contractions, isometric strength produced by the quadricep muscles did not differ between Placebo and Blockade conditions (*p* = 0.89). Isometric strength was reduced immediately following exercise, reaching a maximal reduction (Δ‐19.5 ± 3.4%) at 24‐h after exercise and returned to pre‐exercise levels 72‐h after exercise. There were no differences in strength loss between Placebo and Blockade (*p* = 0.82) nor in the pattern of strength loss between conditions (*p* = 0.91). Similarly, plasma creatine kinase concentrations were not different pre‐exercise between Placebo (62.7 ± 9.9 U/L) and Blockade (63.1 ± 10.5 U/L), and increased above pre‐exercise concentrations at 6, 12, 24, 48 h after exercise (*p* < 0.05). Creatine kinase concentrations increased similarly between Placebo and Blockade conditions (*p* = 0.37) and the pattern of change was not different between groups (*p* = 0.65). By convention, an area under‐the‐curve analysis was performed to represent the extent of damage. The area under‐the‐curve analysis indicated that there was no difference between Placebo and Blockade in the elevation of creatine kinase over 72 h (*p* = 0.29).

#### 3.2.2 Aerobic

During the aerobic exercise protocol, the goal was to have subjects exercise for 60 min at 60% VO_2 peak_. Subjects exercised at 148 ± 12 W. The percentage of heart rate reserve (heart rate reserve is defined as maximal heart rate achieved during VO_2 peak_ testing minus the resting supine heart rate) attained during exercise (Blockade, 63 ± 12%, vs. Placebo, 64 ± 9%; *p* = 0.89) was consistent with the target workload. Heart rate increased from 60 ± 6 beats min^−1^ during rest to 140 ± 15 beats min^−1^ during exercise (measured 20 min into exercise bout) for Blockade and from 54 ± 9 beats min^−1^ during supine rest to 140 ± 11 beats min^−1^ during exercise for Placebo (*p* < 0.05 vs. rest on both days). The percent change in femoral blood flow from pre‐exercise to 60 min post‐exercise tended to be greater for the Placebo (Δ38 ± 22%) compared with Blockade (Δ22 ± 14%; *p* = 0.17). Likewise, the percent change in femoral vascular conductance from pre‐exercise to 60 min post‐exercise tended to be greater for Placebo (Δ46 ± 25%) compared with Blockade (Δ20 ± 13%; *p* = 0.09), consistent with prior studies using this model.

### 3.3 Circulating cell populations

#### 3.3.1 Leukocytes and neutrophils

**FIGURE 2:**
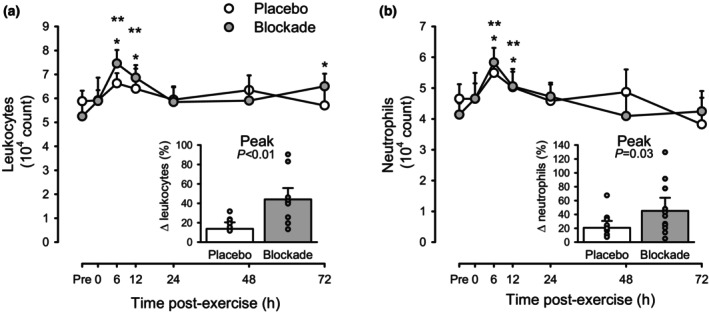
Circulating leukocytes (a) and neutrophils (b) in response to eccentric exercise for placebo (open circles) and histamine‐receptor blockade (closed circles) conditions. Values are means ±95% CI for *n* = 12 (3 female, 9 male). *p < 0.05 versus pre‐exercise within condition and **p < 0.05 versus pre‐exercise across conditions by two‐way repeated‐measures ANOVA with a priori contrasts. Inset bar graphs show the peak response as percent change from pre‐exercise for placebo (open bar) and histamine‐receptor blockade (closed bar).

Figure 2 shows the counts of leukocytes and neutrophils before and across the 72‐h after eccentric exercise. Across conditions (i.e., collapsing the two drug conditions, Placebo and Blockade, to focus on the main effect of time), leukocytes were elevated at 6 and 12 h after exercise (*p* < 0.05 vs. pre‐exercise by ANOVA). However, only in blockade was *p* < 0.05 versus pre‐exercise within a condition. The peak response at 6 h post‐exercise was a 44.1 ± 11.7% increase with Blockade versus 13.7 ± 6.6% with Placebo (both *p* < 0.05 vs. baseline, but also *p* < 0.05 between Blockade and Placebo). Likewise, neutrophils were elevated at 6 and 12 h after exercise (*p* < 0.05 vs. pre‐exercise). However, only in blockade was *p* < 0.05 versus pre‐exercise within a condition. The peak response at 6‐h post‐exercise was a 45.2 ± 19.0% increase with Blockade versus 20.8 ± 10.0% with Placebo (both *p* < 0.05 vs. baseline, but also *p* < 0.05 between Blockade and Placebo).

**FIGURE 3:**
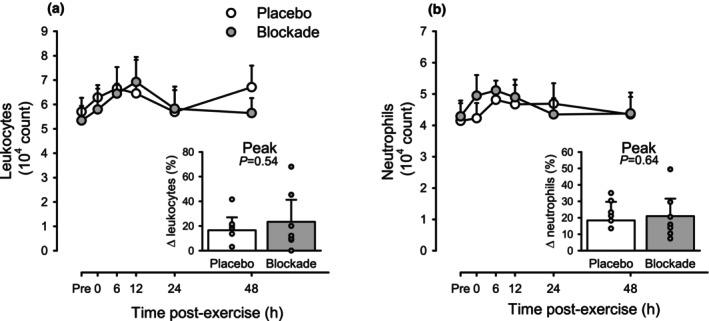
Circulating leukocytes (a) and neutrophils (b) in response to aerobic exercise for placebo (open circles) and histamine‐receptor blockade (closed circles) conditions. Values are means ±95% CI for *n* = 7 (2 female, 5 male). No differences detected by two‐way repeated‐measures ANOVA. Inset bar graphs show the peak response as percent change from pre‐exercise for placebo (open bar) and histamine‐receptor blockade (closed bar). No differences detected between blockade and placebo by paired *t*‐test.

Figure 3 shows the counts of leukocytes and neutrophils before and across the 48 h after aerobic exercise. Across conditions (i.e., collapsing the two drug conditions), leukocytes tended to be elevated across time after exercise (*p* = 0.07 vs. pre‐exercise). The peak response at 6‐h post‐exercise was a 23.4 ± 17.9% increase with Blockade versus 16.5 ± 10.5% with Placebo (both *p* < 0.05 vs. baseline, *p* = 0.54 between Blockade and Placebo). Likewise, neutrophils were not clearly elevated across time after exercise (*p* = 0.65 vs. pre‐exercise). The peak response at 6‐h post‐exercise was a 21.0 ± 10.6% increase with Blockade versus 18.4 ± 11.4% with Placebo (both *p* < 0.05 vs. baseline, *p* = 0.64 between Blockade and Placebo).

#### 3.3.2 Other cell populations

Table 2 shows the percentage of leukocytes that represented various cell populations before and across the 72 h after eccentric exercise. Across conditions (i.e., collapsing the two drug conditions), MON1 and angiogenic monocytes were reduced at 48 and 72 h after exercise (*p* < 0.05 vs. pre‐exercise by ANOVA) due to reductions in both Blockade and Placebo conditions (which did not differ). Granulocytes, MON2, MON3, and endothelial progenitor cell types did not differ across time.

**TABLE 2:** Circulating cell populations and eccentric exercise.

Time post‐exercise (h)	Pre	0	6	12	24	48	72	Condition
Granulocytes (% total)	41.3 ± 8.1	45.5 ± 6.4	47.3 ± 11.9	49.5 ± 6.8	43.5 ± 7.2	45.3 ± 10.0	37 ± 8.4	Placebo
	41.2 ± 3.0	47.7 ± 8.8	57.4 ± 4.4	50.5 ± 6.4	43.2 ± 7.7	40.9 ± 9.8	37.9 ± 5.8	Blockade
Endothelial progenitor cells (% total)	1.1 ± 0.3	3.3 ± 3.1	1.7 ± 0.9	1.5 ± 0.4	1.5 ± 0.5	1.5 ± 0.6	1.4 ± 0.5	Placebo
	0.9 ± 0.3	1.3 ± 0.6	0.8 ± 0.2	4.7 ± 5.9	1.0 ± 0.2	1.3 ± 0.3	1.0 ± 0.3	Blockade
Mon1 (% total)	2.3 ± 0.8	2.1 ± 0.6	2.2 ± 0.6	2.2 ± 1.0	2.0 ± 0.7	1.5 ± 0.8* ^,^ **	1.1 ± 0.4* ^,^ **	Placebo
	2.5 ± 0.6	1.9 ± 0.7	2.3 ± 0.5	2.1 ± 0.5	2.0 ± 0.6	2.0 ± 0.6**	1.4 ± 0.4* ^,^ **	Blockade
Mon2 (% total)	0.6 ± 0.4	0.2 ± 0.1	0.6 ± 0.5	0.1 ± 0.1	0.2 ± 0.2	0.3 ± 0.2	0.3 ± 0.1	Placebo
	0.1 ± 0.1	0.1 ± 0.1	0.2 ± 0.3	0.3 ± 0.5	0.3 ± 0.3	0.3 ± 0.2	0.2 ± 0.2	Blockade
Mon3 (% total)	0.1 ± 0.0	0.1 ± 0.1	0.6 ± 0.8	0.2 ± 0.1	0.1 ± 0.1	0.2 ± 0.3	0.1 ± 0.0	Placebo
	0.1 ± 0.0	0.2 ± 0.2	0.1 ± 0.1	0.1 ± 0.1	0.1 ± 0.0	0.1 ± 0.1	0.1 ± 0.0	Blockade
Angiogenic monocyte (% total)	2.8 ± 0.7	2.3 ± 0.6	2.6 ± 0.7	2.2 ± 0.9	2.1 ± 0.6	1.8 ± 0.9* ^,^ **	1.3 ± 0.5* ^,^ **	Placebo
	2.5 ± 0.6	2.1 ± 0.6	2.4 ± 0.5	2.1 ± 0.5	2.2 ± 0.4	2.2 ± 0.6**	1.3 ± 0.3* ^,^ **	Blockade

*Note*: Circulating cell populations in response to eccentric exercise for placebo and histamine‐receptor blockade conditions. Values are mean ± 95% CI for *n* = 12 (3 female, 9 male).

* *p <* 0.05 versus pre‐exercise within condition by two‐way repeated‐measures ANOVA with a priori contrasts.

** *p <* 0.05 versus pre‐exercise across conditions by two‐way repeated‐measures ANOVA with a priori contrasts. No differences detected between blockade and placebo.

Table 3 shows the percentage of leukocytes that represented various cell populations before and across the 48 h after aerobic exercise. Granulocytes, endothelial progenitor cells, and monocyte subpopulations did not differ across time.

**TABLE 3:** Circulating cell populations and aerobic exercise.

Time post‐exercise (h)	Pre	0	6	12	24	48	Condition
Granulocytes (% total)	39.3 ± 14.0	44.6 ± 8.0	41.0 ± 10.3	43.2 ± 8.6	46.8 ± 7.1	41.5 ± 7.4	Placebo
	42.0 ± 8.0	52.5 ± 7.3	49.0 ± 14.5	50.0 ± 8.9	44.9 ± 12.5	45.4 ± 10.7	Blockade
Endothelial progenitor cells (% total)	3.9 ± 3.2	1.5 ± 1.2	2.2 ± 1.2	2.4 ± 1.2	1.5 ± 0.4	1.4 ± 0.5	Placebo
	1.6 ± 0.8	1.9 ± 1.2	1.4 ± 1.1	1.0 ± 0.6	1.5 ± 0.8	2.0 ± 1.6	Blockade
Mon1 (% total)	0.6 ± 0.4	1.7 ± 1.2	4.2 ± 5.2	1.4 ± 1.1	1.5 ± 1.5	1.1 ± 1.2	Placebo
	1.7 ± 0.8	0.9 ± 0.3	0.8 ± 0.3	1.4 ± 0.9	1.7 ± 1.0	1.5 ± 0.8	Blockade
Mon2 (% total)	0.2 ± 0.2	0.1 ± 0.1	0.2 ± 0.2	0.1 ± 0.1	0.6 ± 0.4	0.3 ± 0.2	Placebo
	0.1 ± 0.1	0.1 ± 0.1	0.3 ± 0.3	0.2 ± 0.2	0.1 ± 0.0	0.3 ± 0.3	Blockade
Mon3 (% total)	0.2 ± 0.3	0.3 ± 0.1	0.2 ± 0.1	0.2 ± 0.2	0.2 ± 0.2	0.2 ± 0.1	Placebo
	0.2 ± 0.1	0.2 ± 0.2	0.1 ± 0.1	0.1 ± 0.0	0.2 ± 0.1	0.2 ± 0.2	Blockade
Angiogenic monocyte (% total)	0.9 ± 0.5	2.0 ± 1.3	1.4 ± 0.8	1.6 ± 0.8	1.8 ± 1.1	1.6 ± 1.1	Placebo
	1.5 ± 0.6	1.2 ± 0.4	1.1 ± 0.3	1.4 ± 0.8	1.6 ± 0.8	1.7 ± 0.8	Blockade

*Note*: Circulating cell populations in response to aerobic exercise for placebo and histamine‐receptor blockade conditions. Values are mean ± 95% CI for *n* = 7 (2 female, 5 male). No differences detected by two‐way repeated‐measures ANOVA.

### 3.4 Circulating cytokines

**FIGURE 4:**
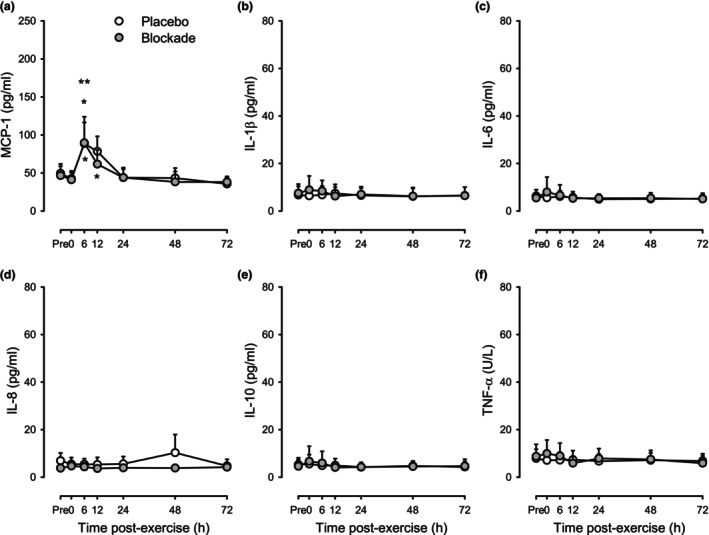
Circulating cytokines in response to eccentric exercise for placebo (open circles) and histamine‐receptor blockade (closed circles) conditions. (a) MCP‐1, (b) IL‐1β, (c) IL‐6, (d) IL‐8, (e) IL‐10, and (f) TNF‐α. Values are means ±95% CI for *n* = 12 (3 female, 9 male). *p < 0.05 versus pre‐exercise within condition and **p < 0.05 versus pre‐exercise across conditions by two‐way repeated‐measures ANOVA with a priori contrasts. No differences detected between blockade and placebo.

Figure 4 shows the concentrations of MCP‐1, IL‐1β, IL‐6, IL‐8, IL‐10, and TNF‐α before and across the 72 h after eccentric exercise. Across conditions (i.e., collapsing the two drug conditions), only MCP‐1 showed elevations at 6 and 12 h after exercise (*p* < 0.05 vs. pre‐exercise by ANOVA). The peak response at 6‐h post‐exercise was a 104.0 ± 72.5% increase with Blockade versus 93.1 ± 41.9% with Placebo (both *p* < 0.05 vs. baseline, *p* = 0.82 between Blockade and Placebo).

**FIGURE 5:**
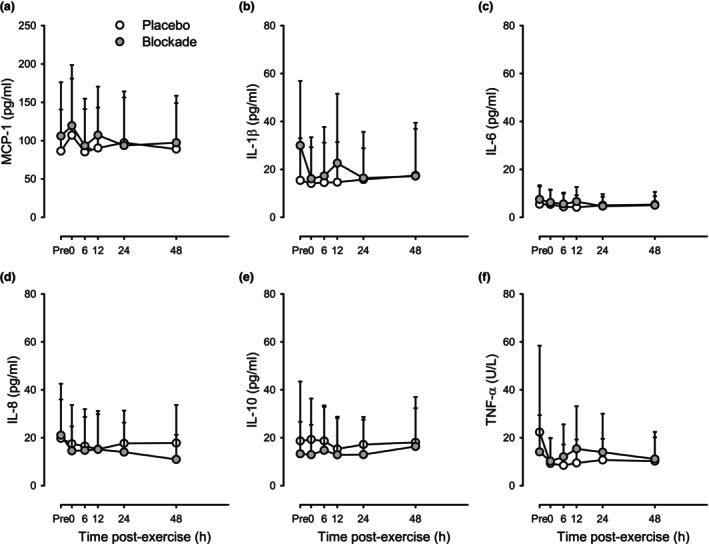
Circulating cytokines in response to aerobic exercise for placebo (open circles) and histamine‐receptor blockade (closed circles) conditions. (a) MCP‐1, (b) IL‐1β, (c) IL‐6, (d) IL‐8, (e) IL‐10, and (f) TNF‐α. Values are means ±95% CI for *n* = 5 (1 female, 4 male). No differences detected by two‐way repeated‐measures ANOVA.

Figure 5 shows the concentrations of MCP‐1, IL‐1β, IL‐6, IL‐8, IL‐10, and TNF‐α before and across the 48 h after aerobic exercise. No differences were detected by two‐way repeated‐measures ANOVA.

As MCP‐1 was the only inflammatory marker that appeared to have changed following exercise, we used correlation analysis to determine if changes in MCP‐1 at 6‐h post‐exercise were associated with the changes in circulating cell populations at that time point. As indicated in Table 4, the rise in MCP‐1 after eccentric exercise was inversely related to changes in endothelial progenitor cells and the rise in MCP‐1 after aerobic exercise was inversely related to changes in leukocytes. It is unclear whether these are meaningful relations or spurious associations.

**TABLE 4:** Correlations between changes in MCP‐1 and circulating cell populations.

Exercise	Eccentric		Aerobic	
	*r*	*p*‐value	*r*	*p*‐value
Leukocytes	−0.306	0.15	−0.650	0.04*
Neutrophils	−0.082	0.70	0.144	0.69
Granulocytes	−0.129	0.55	−0.036	0.92
Endothelial progenitor cells	−0.481	0.02*	0.063	0.86
Mon1	−0.037	0.86	0.538	0.11
Mon2	0.382	0.07	0.637	0.07
Mon3	−0.297	0.17	−0.348	0.40
Angiogenic monocyte	−0.115	0.59	−0.008	0.98

*Note*: Pearson correlation (*r*) and corresponding *p*‐values for the change in circulating cell populations versus the change in MCP‐1 from pre‐exercise to 6‐h post‐exercise in response to eccentric exercise and aerobic exercise for *n* = 24 Eccentric (3 female, 9 male, times 2 conditions) and *n* = 10 aerobic (1 female, 4 male, times 2 conditions).

* *p* < 0.05.

## 4 DISCUSSION

This is the first known investigation to examine the link between histamine and the systemic inflammatory response following exercise. The main findings of the present investigation were that taking combined histamine H_1_ and H_2_ receptor antagonists prior to eccentric exercise increased the magnitude but not the duration of the increase of circulating leukocytes and neutrophils that occurred following this muscle damaging exercise and had negligible influences on circulating cytokines. A smaller rise in magnitude of leukocytes and neutrophils was observed following the 60 min of moderate intensity aerobic exercise but this rise was not affected by histamine blockade. These findings were counter to our hypothesis and our expectations, but this response pattern is compatible with our previous observations of (1) a substantial rise of intramuscular histamine during exercise (Romero, McCord, et al., 2016b), (2) a histamine‐mediated elevation of skeletal muscle blood flow following exercise that last hours (McCord et al., 2006; McCord & Halliwill, 2006), (3) an increase in expression of immune function genes following exercise that is altered with histamine blockade (Romero et al., 2018). These observations may link with the many associations between histamine and inflammatory responses outside of exercise (Nettis et al., 2005; Noli & Miolo, 2001; Numata et al., 2006; Yoshida et al., 1989).

### 4.1 Exercise inflammatory response

It is now generally accepted that a tightly regulated inflammatory response is necessary for muscle repair and regeneration following strenuous exercise (Peake et al., 2017; Tidball, 2005). Multiple inflammatory cell types including mast cells, neutrophils, and monocytes aid in this response (MacIntyre et al., 1995, 1996; Marklund et al., 2013; Peake et al., 2017; Tidball & Villalta, 2010; Tidball & Wehling‐Henricks, 2007). Following exercise, there is an immediate and robust increase in circulating leukocytes that wanes in the hours to days that follow (Campbell & Turner, 2018; Hansen et al., 1991; Nieman et al., 2005; Peake et al., 2005; Van Craenenbroeck et al., 2014). Within an hour after exercise, the circulating neutrophils are observed in the extracellular space of skeletal muscle tissue and monocytes/macrophages appear in the following hours to days (Campbell & Turner, 2018; Deyhle et al., 2015; MacIntyre et al., 1996; Peake et al., 2017). There are also increases in circulating cytokines, many released from the contracting muscle, that are part of a paracrine and endocrine communication for the coordination of the inflammatory response (Peake et al., 2015; Pedersen & Febbraio, 2008). Once inside the muscle tissue, the neutrophils and monocytes/macrophages breakdown damaged tissue and begin the repair process. Although the leukocytes and cytokines aid in inflammatory response after exercise, many aspects in the mobilization, regulation, of repair of tissue are unclear and involve unknown molecules (Peake et al., 2017).

### 4.2 Histamine

Histamine is a molecule that not only has associations to immune and inflammatory responses but is also released and produced within the contracting skeletal muscle. Intramuscular histamine concentrations increase ~3 fold during moderate intensity endurance exercise (Romero, McCord, et al., 2016b). It is possible that the exercise‐induced rise in intramuscular histamine is involved with the exercise inflammatory response in order to deliver and direct the circulating leukocytes to the muscle tissue. For example, in immune and inflammatory responses, histamine aids in the diapedesis of leukocytes from circulation into damaged/infected tissue. The diapedesis occurs through a histamine‐initiated margination of capillary endothelial and pericyte cells, an increased capillary permeability, the expression of adhesion molecules on endothelial cells, and in acting as a chemoattractant to leukocytes (Beer et al., 1984; Jutel et al., 2002; Packard & Khan, 2003). Histamine may also be involved with the delivery of immune cells to the muscle as there is a histamine mediated dilation of resistance arterioles that result in an elevation of local blood flow that can last upward of 120 min within the previously active muscle (McCord et al., 2006; McCord & Halliwill, 2006; Romero, McCord, et al., 2016b). Further evidence that histamine is involved with the exercise inflammatory response comes from a recent study that documented a down regulation of approximately 800 protein‐coding genes within muscle tissue with a systemic blocking of histamine actions. Many of the protein‐coding genes were related to inflammation and cell maintenance (Romero et al., 2018; Romero, Hocker, et al., 2016a). Therefore, histamine may be a link between exercise and the associated inflammatory response.

### 4.3 Inflammatory response

Similar to previous reports of muscle damaging exercise, in the present study there was a transient elevation in circulating leukocytes that peaked 6 h after eccentric exercise and returned to baseline levels approximately 24 h after exercise (MacIntyre et al., 1996; Peake et al., 2005, 2017) (Figure 2). A smaller trend for increased leukocyte count was also observed 6 h following aerobic exercise (Figure 3). It was hypothesized that exercise‐associated elevation of histamine would aid in the migration of leukocytes out of circulation and into the previously active skeletal muscle. This hypothesis was based on the functions of histamine during inflammation, characterized by the vasodilation of blood vessels, increased capillary permeability, and expression of endothelial adhesion molecules. Therefore, blocking histaminergic actions during and following exercise might result in an extended elevation in leukocyte concentrations. Contrary to this hypothesis, blocking histamine's actions augmented the magnitude of leukocytosis following eccentric exercise but not the time‐course (Figure 2). It is unknown if the skeletal muscle infiltration of the leukocytes was reduced in Blockade versus Placebo conditions. It is possible that the entry of leukocytes into the skeletal muscle was impeded and that this process involved a sufficiently large quantity of cells to impact circulating levels. It is also possible that histamine receptor antagonism alters the entry of leukocytes into the circulation; however, we speculate that since circulating cytokines appeared unaffected by antagonism, this is the less likely explanation.

### 4.4 Monocytes

Studies indicate that the leukocytosis after exercise is not limited to one cell type and many cell types may increase in different proportions (Hoffman‐Goetz et al., 1990). In addition, cell culture experiments with polymorphic mononuclear cells suggest that histamine has the ability to increase the expression of interleukin‐10 and interleukin‐12 (Jutel et al., 2001; Packard & Khan, 2003). These two interleukins control the expression of cell surface markers and function of monocyte subpopulations (Jutel et al., 2001; Packard & Khan, 2003). Therefore, it was hypothesized that blocking histamine during exercise may also alter the percentage of monocyte sub‐populations within the leukocyte response. Contrary to this hypothesis, neither the percentage of monocytes nor the subpopulation of monocytes were altered over the 48–72 h of recovery from aerobic or muscle damaging exercise by the administration of histamine receptor antagonists.

### 4.5 Cytokines

Immediately after exercise, there is an increased concentration of circulating cytokines such as IL‐6, TNFa, IL‐1B, MCP‐, IL‐8, IL‐1 RA, and IL‐10 (Chen et al., 2014; Field et al., 1991; Nieman et al., 2005; Pedersen & Febbraio, 2008; Smith et al., 2000). In the present study, there was an elevation in MCP‐1 following eccentric exercise (Figure 4), but no detectable difference with the administration of histamine receptor antagonists. This chemoattractant protein is produced by endothelial and skeletal muscle myocytes during contractions and is involved with monocyte recruitment into tissues (Della‐Gatta et al., 2014; Van Craenenbroeck et al., 2014).

### 4.6 Exercise mode

One cross‐sectional study examining the acute inflammatory period (<6 h) following exercise found some differences in the rise in leukocytes between maximal aerobic exercise (>90% VO_2_ for 5 min), prolonged aerobic exercise (60% VO_2_ for 2 h), or resistance exercise (Natale et al., 2003). Additionally, an acute elevation of histamine related cytokines (IL‐6 and TNF‐a) was only observed following prolonged aerobic exercise (Brenner et al., 1999). Thus, while not our primary goal, we note the general similarities of the additional data in the present study on aerobic exercise compared to eccentric exercise, in that both show patterns of elevated leukocytosis that resolves in ~24 h. This is quicker than we had anticipated for eccentric exercise, which we thought would produce a more prolonged a leukocytosis compared to aerobic exercise (Brenner et al., 1999; Peake et al., 2017). However, the contribution of histamine from skeletal muscle may vary in magnitude between these two models such that the systemic effects are not obvious with aerobic exercise of moderate intensity. We do not try to over‐interpret these observations.

### 4.7 Limitations

The present study was limited in that it only examined the systemic inflammatory response and did not examine the skeletal muscle directly. Histamine concentrations are increased within the skeletal muscle during repeated contractions (Romero, McCord, et al., 2016b) and are reduced by rapid breakdown through membrane bound and cytosolic enzymes (Ind et al., 1982). Due to the rapid breakdown of histamine, it has a short half‐life (~100 s) and any physiological actions of histamine are likely in close proximity to where it is released/produced. If histamine is participating in the exercise associated inflammatory response, it is likely at the level of the endothelial, pericyte, mast, or other associated cells within the muscle tissue. If this is a reasonable interpretation of the current data and known functions of histamine, then the skeletal muscle tissue should be the focus of follow up examinations. Additionally, as noted in the methods, we had to replace the H_2_ antagonist that was being used part way through the aerobic exercise cohort, and ultimately ceased data collection due to the pandemic.

### 4.8 Conclusion

The present findings address important issues identified by The National Institutes of Health (NIH) on the interaction of physical activity, inflammation, and immune function (Neufer et al., 2015). It guides the scientific understanding of the exercise‐induced rise in skeletal muscle histamine and its association with inflammation resulting from muscle damaging exercise. Histamine is a molecule most associated with inflammatory and immune responses. The discovery that histamine concentrations are increased within skeletal muscle during exercise has led to a number of intriguing observations, including the key finding that histamine had a broad impact on the exercise transcriptome (Romero et al., 2018; Romero, Hocker, et al., 2016a) and is critical to the robust adaptations to exercise training (Van der Stede et al., 2021). The idea, that exercise initiates a pro‐inflammatory state, suggests an important role for histamine in this process. It may be that the inflammatory signaling initiated by histamine is a key feature of how humans adapt to exercise. The present study supports the idea that histaminergic signaling within skeletal muscle can impact leukocytosis in response to muscle‐damaging exercise, suggesting that histamine is exerting a local influence within the skeletal muscle that can impact systemic responses to exercise.

## AUTHOR CONTRIBUTIONS

MRE: Conceived and designed the experiment, conceived and designed the experiment, analyzed data, interpreted results of experiments, prepared figures, drafted manuscript, edited and revised manuscript, and approved final version of manuscript. JEM: Conceived and designed the experiment, conceived and designed the experiment, analyzed data, interpreted results of experiments, prepared figures, drafted manuscript, edited and revised manuscript, and approved final version of manuscript. KWN: Conceived and designed the experiment, analyzed data, interpreted results of experiments, prepared figures, drafted manuscript, edited and revised manuscript, and approved final version of manuscript. CTM: Conceived and designed the experiment, interpreted results of experiments, edited and revised manuscript, and approved final version of manuscript. JRH: Conceived and designed the experiment, analyzed data, interpreted results of experiments, prepared figures, drafted manuscript, edited and revised manuscript, and approved final version of manuscript.

## FUNDING INFORMATION

This research was supported in part by National Institutes of Health Grants AG072805 and HL115027, American Heart Association Grant 17GRNT33660656, and the Eugene and Clarissa Evonuk Memorial Graduate Fellowship.

## CONFLICT OF INTEREST STATEMENT

The authors have no conflicts of interests, financial or otherwise, that would be affected by the outcome of this publication.

## ETHICS STATEMENT

This study was approved by the Institutional Review Board at the University of Oregon in accordance with the Declaration of Helsinki, with the exception of registration of a database. All participants provided written informed consent after being briefed on all aspects of the experimental protocol, including the risks.

## Data Availability

The data that support the findings of this study are available on request from the corresponding author. The data are not publicly available due to privacy or ethical restrictions.
